# Genetic evidence for panmixia in a colony-breeding crater lake cichlid fish

**DOI:** 10.1038/s41598-018-19266-5

**Published:** 2018-01-18

**Authors:** T. K. Lehtonen, K. R. Elmer, M. Lappalainen, A. Meyer

**Affiliations:** 1 Zoology and Evolutionary Biology, Department of Biology, University of Konstanz, 78457 Konstanz, Germany; 2 School of Biological Sciences, Monash University, Victoria, 3800 Australia; 3 Institute of Biodiversity, Animal Health & Comparative Medicine, College of Medical, Veterinary & Life Sciences, University of Glasgow, Glasgow, G12 8QQ UK

## Abstract

Fine-scaled genetic structuring, as seen for example in many lacustrine fish, typically relates to the patterns of migration, habitat use, mating system or other ecological factors. Because the same processes can also affect the propensity of population differentiation and divergence, assessments of species from rapidly speciating clades, or with particularly interesting ecological traits, can be especially insightful. For this study, we assessed the spatial genetic relationships, including the genetic evidence for sex-biased dispersal, in a colony-breeding cichlid fish, *Amphilophus astorquii*, endemic to Crater Lake Apoyo in Nicaragua, using 11 polymorphic microsatellite loci (n = 123 individuals from three colonies). We found no population structure in *A*. *astorquii* either within colonies (no spatial genetic autocorrelation, r ~0), or at the lake-wide level (pairwise population differentiation *F*_ST_ = 0–0.013 and no clustering), and there was no sex-bias (male and female AIc values bounded 0) to this lack of genetic structure. These patterns may be driven by the colony-breeding reproductive behaviour of *A*. *astorquii*. The results suggest that strong philopatry or spatial assortative mating are unlikely to explain the rapid speciation processes associated with the history of this species in Lake Apoyo.

## Introduction

Dispersal, when resulting in gene flow, is a fundamental process in evolution. In interaction with other behavioural characteristics and demography, it can exert a large influence on genetic population structure^1,2^. Molecular methods have revealed very fine scaled genetic structuring among many animal populations, especially those of lacustrine fish^3–7^. These patterns often result from low levels of migration, a lack of significant larval dispersal, and a tendency for philopatry by the adults. Interestingly, high levels of such philopatric behaviour have been found across a wide range of taxa, including insects, fish, amphibians, birds and mammals^8–10^.

Patterns of population structure may also relate to habitat use and feeding preferences, with trophic and habitat specialists often exhibiting more restricted gene flow over any habitat discontinuities than more opportunistic species^4,11–13^. However, such ecological barriers are not necessarily required to induce philopatric behaviour^14,15^. For example, in *Variabilichromis moorii*, a monogamous Lake Tanganyikan cichlid fish, mitochondrial DNA sequences indicated that dispersal is very infrequent and at similar levels along continuous stretches of rocky shore and across localities separated by sandy habitat^14^. Dispersal patterns affecting the population genetic structure can also be sex specific. For example, in superb fairy-wrens, *Malurus cyaneus*, that exhibit fine-scale genetic structure, any male dispersal from their natal territory occurs only over very short distances, whereas female fairy-wrens show also relatively long distance dispersal^2,16^. In cichlid fish of Lake Malawi, in turn, gene flow occurs primarily by dispersal of sub-adult males that search for breeding territories, with females tending to stay in their natal area^15,17^. As a result, kin structuring of the population may be evident only within the philopatric sex, typically males in birds and females in mammals and fish^8^. Such sex-biased dispersal may be related to avoidance of the risk of inbreeding, competition with kin, sex biased co-operation with kin, or sex specific territorial behaviour^8,18–21^. More generally, mating system characteristics, such as the patterns of territoriality and mating competition, can markedly contribute to the genetic structure^15,22–25^.

Factors that affect dispersal and smaller scale genetic structure can also influence the likelihood of speciation^26–31^. This is especially true for philopatry: for example the outstandingly species rich radiations of cichlid fish in the African Great Lakes have been associated with philopatric tendencies, as well as genetic differentiation over short distances within the lakes^3,4,14^. Researchers have also suggested that groups with a greater population genetic structure should be more diverse than clades without fine-scale structure, as reduction in gene flow may be linked to isolation^12^. On the other hand, lacustrine fish are also known to be particularly prone to speciate in sympatry^32^, with pronounced differences in speciation rates between even closely related clades^12,33^. Therefore, to investigate the importance of different speciation mechanisms, studies on the present genetic structure in clades that are exceptionally fast (or slow) to speciate can be particularly informative^12,34,35^.

In this study, we assessed the population structure at different spatial scales, as well as the genetic evidence for sex-biased dispersal, in a cichlid fish, *Amphilophus astorquii*, which is endemic to Crater Lake Apoyo in Nicaragua. *Amphilophus astorquii* was chosen for the study for multiple reasons. First, it is a species from a particularly rapidly speciating cichlid clade that inhabits mostly Nicaraguan freshwaters, with six endemic species of the group living in Lake Apoyo alone^33,36,37^. Indeed, similarly to its congeners in Nicaraguan crater lakes, *A*. *astorquii* seems to have evolved through sympatric speciation^38,39^. Second, the species is notable for breeding in dense breeding aggregations, while also showing high levels of territorial competition with its conspecific, and occasionally heterospecific, neighbours^40^. Third, it has a more benthic life style than *Amphilophus zaliosus*, a closely related and sympatric pelagic species^38^. In this regard, pelagic cichlids are often thought to have less population structure than those using more benthic habitats^13,41,42^. Finally, at the time of the study, *A*. *astorquii* was locally the most abundant breeding cichlid species^40,43^. Such colony-like breeding aggregations allowed sampling at different levels of physical distance, in order to make interferences about migration and gene flow within the lake. Specifically we tested: (a) whether there is genetic differentiation between breeding colonies, and if so, whether such patterns are sex-biased, either of which would suggest site fidelity in the scale of the whole lake; and (b) whether there is a spatial pattern of relatedness within colonies, which would suggest fine-scale site fidelity and/or kin-grouping influencing spatial distributions.

## Materials and Methods

The sampling was conducted in Lake Apoyo. The crater lake formed approximately 23 000 years ago^44^ and is the largest of its kind in Nicaragua, with diameter of ~5 km, shoreline of ~20 km and maximum depth of 175 m^45^. Our model species, *A*. *astorquii*, is endemic to the lake, where it arose by sympatric speciation from a generalist ancestor^38,46^. It is the most distinctly colony-breeding species in the Midas cichlid complex, in which males and females are thought to pair monogamously around the same time they claim a territory^47^ and then provide parental care until their fry are approximately six weeks old. The fish may survive to breed during more than one breeding season (with the same or a new social partner).

**Figure 1:**
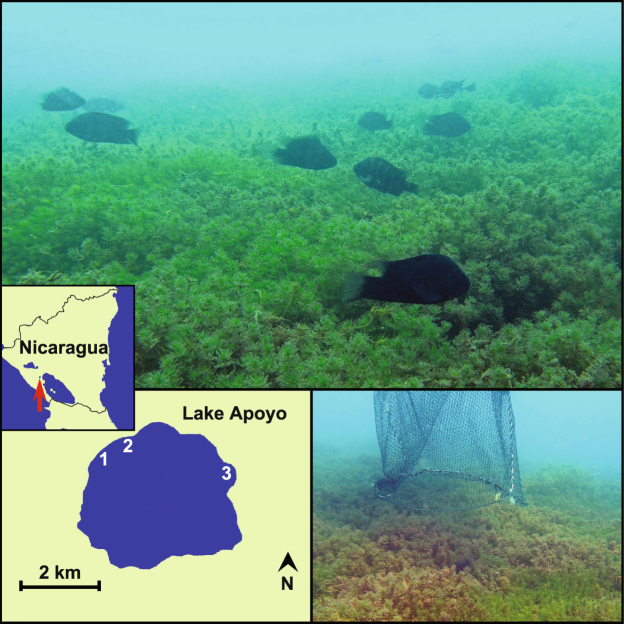
Colony 1 amidst a thick bed of *Chara* algae (top section, photo taken by TKL on 18/December/2007), outline of Lake Apoyo with locations of the sampled *Amphilophus astorquii* colonies (bottom left, drawn by TKL in Adobe Photoshop Elements 4.0, http://www.adobe.com/products/photoshop-elements.html), a sampling net that was quickly placed over a pair seeking shelter within *Chara* (bottom right, photo taken by TKL on 13/December/2007).

Aggregations of *A*. *astorquii* breeding pairs, from here on referred to as ‘colonies’ (defined as an area of a high concentration of breeding pairs with no, or very few, breeding individuals in the intercepting area between colonies) within Lake Apoyo were associated with beds of *Chara* algae. *Chara* does not occur everywhere in the lake, contributing to the patchiness of different habitat types (i.e. sandy and predominantly rocky areas with and without *Chara*). The colonies were the densest at depths of 4–8 metres, with each pair maintaining a territory of approximately 0.5 m diameter, this also being the minimum distance between adjoining territories (Fig. 1).

### Sampling fishes

Several locations around the lake were pre-surveyed within the limitations of time constraints, divers’ access to the lake from the shore and reasonable dive/swim times from these access points. Five to six colonies were identified, and logistic limitations allowed sampling of three representative colonies (Fig. 1) that were both accessible and large enough for our sampling procedure (see below for details). The samples were collected in December 2007, during the peak of the breeding season, which in this species usually lasts from November to January^40,48^. All colonies were associated with thick *Chara* beds (Fig. 1) in areas of gentle to moderately steep depth slopes at depths of 3.5–8 metres. Prior to sampling, SCUBA divers set a transect line, roughly parallel to the shore line, through each colony. Social pairs (males and females) were then collected from 3 m intervals along the full length of each transect, using hand nets (Fig. 1), with harpoons as a back-up. When approached, the pair withdrew to the centre of their territory within *Chara* growth (Fig. 1). The net was then rapidly pushed against the substratum, capturing the pair. In total, 123 adult individuals were collected and genotyped from the three colonies (Fig. 1): Colony 1 n* = *63 (30 pairs, 2 females, 1 male); Colony 2 n = 23 (10 pairs, 2 females, 1 male); Colony 3 n = 37 (16 pairs, 3 females, 2 males).

### Genotyping

Genomic DNA was isolated from ethanol-preserved tissue using standard proteinase K digestion followed by high salt extraction methods, resuspended in water and stored at −20 °C. Eleven microsatellite loci were amplified using standard PCR conditions: Abur151, Abur82, Abur28, Abur45^49^, M1M, M2, M7, M12^50^, Unh012, Unh013^51^, and TmoM7^52^. Ten percent of individuals were repeated as technical replicates. For each locus, one primer was labelled with a NED, FAM or HEX fluorescent dye (Applied Biosystems) for subsequent visualization. Loci were genotyped in an ABI 3130xl (Applied Biosystems) and sized according to Rox standard (Applied Biosystems).

Genotyping was quality controlled for errors and null alleles in Micro-checker^53^. Analyses of all samples in a single population suggested a low presence of null alleles (excess homozygosity) at Abur 28 (0.06 Brookfield frequency in homozygotes), Abur45 (0.17), and Unh012 (0.14)^54^. However this signal was not found when each colony was tested separately (i.e. in no case did each colony have null alleles inferred by Micro-checker), suggesting it is not a pervasive bias in the data and so all loci were retained. To verify the inferred pattern, analyses that might be particularly sensitive to homozygosity were also conducted using a reduced number of loci (see Results). Data are available in Table S3.

### Population genetic analyses

Summary statistics of diversity and population structure were calculated in GenoDive v2.0b27^55^ and statistical significance assessed with 9999 permutations. Infinite allele model was assumed for calculating the analysis of molecular variance (AMOVA). *F*_IS_ statistics were calculated in GenePop on the Web v4.2 (Markov chain parameters: dememorization 1000, batches 100, iterations per batch 1000).

Spatial genetic autocorrelation was assessed over the three ‘populations’ in Genalex v 6.501^56^. Genetic and geographic distance matrices were calculated for each of the three colonies using 11 loci and significance assessed with 999 permutations. Distance classes were set at 3 (for the 3 m intervals sampled along transects from 0 to 102 m) and the number of distance classes set at 30.

Genetic variation was visualised with an individuals-based Principal Coordinates Analysis (PCoA) of genotypic distances (co-variance-standardized method) in GenAlEx. A genetic clustering analysis was conducted in Structure v 2.4.3^57^, with model setting for correlated allele frequencies and admixture among populations without population information (no pop flag), run for 600,000 MCMC generations after 100,000 generations burning. Six replicates were run for K = 1 through K = 4, and log-likelihood results plotted in Structure Harvester^58^.

Sex-biased dispersal was tested in Genalex. As that test tolerates no missing genotypes, we replaced any missing data with the two most common alleles at that locus (0 to 19 individuals across loci) and removed one individual and one locus (TmoM7), with a higher proportion of missing data, in colony 3.

MARENA Nicaragua issued the necessary permits for the study (permit numbers 026/-11007/DGAP and DGRNB-IC-006-2007) and it was performed in accordance with the laws of Nicaragua.

### Data availability

Data are made available in Table S3.

## Results

### Summary statistics

Observed heterozygosity was 0.510 ± 0.073 and heterozygosity within populations 0.576 ± 0.073 (Table S1). Based on the full microsatellite dataset of 11 loci, analysing the lake as a population resulted in deviation from HWE (Chi^2^ = infinity, Df = 62, Prob = High sign). Each colony also deviated significantly from HWE when assessed separately, though there was no strong pattern across loci and colonies, with the exception of high *F*_IS_ in Unh012 for all colonies (Table S2).

### Population differentiation

**Figure 2:**
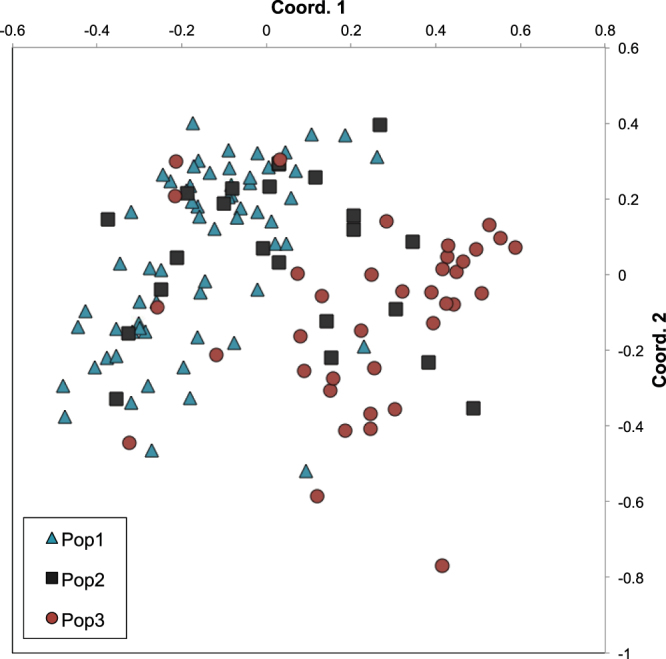
Principal coordinates analysis of individual variation visualised on the first and second axis, including all *A*. *astorquii* from colonies (populations) 1, 2, and 3.

An *F*-statistic-based AMOVA analysis suggested there was little population genetic differentiation among colonies, with inter-population variation contributing less than 1% of the total variation (Table 1). This inference is robust to locus-specific deviations from HWE, as a reduced analysis excluding loci with significant deviations (excluding Abur28, Abur45, Unh012) yielded the same AMOVA results (97.3% variation among individuals, 2.1% among individuals within populations, 0.6% among populations; *F*_ST_ = 0.006, p = 0.034). The PCoA plot (axis 1 vs axis 2) shows some weak separation along coordinate 1 between colony 1 and 2 vs. colony 3, but considerable overlap of individuals from different colonies (Fig. 2). A Structure analysis to infer genetic clusters suggests *K* = 1 as the most likely genetic grouping, with any other grouping (K > 1) being poorly supported, with lower likelihood and higher standard deviation (Fig. S1). Visualisation of the admixture proportions for all individuals at more than one group showed no clustering, with all individuals being equally admixed to two or three groups (Q ~ 50% or 33% for each individual; not shown).

**Table 1:** Analysis of molecular variance based on infinite allele model, across individuals and colonies with F-statistics corresponding to Weir and Cockerham^70^.

Source of Variation	%var		F-value	±SD	P
Within Individual	90.8%	*F*_IT_	0.092	0.034	—
Among Individual Within populations	8.4%	*F*_IS_	0.085	0.034	0
Among Population	0.7%	*F*_ST_	0.007	0.005	0.011

Population genetic differentiation from a pairwise analysis between colonies was also low, with *F*_ST_ between populations ranging from 0 to 0.013, in agreement with the findings from the AMOVA analysis. With permutation there was significant differentiation between Colony 1 and Colony 3 (Table 2), though the absolute values were low. If loci deviating significantly from HWE (Abur28, Abur45, Unh012) were excluded from the analysis, the population differentiation between colonies 1 and 3 was not significant based on the log-likelihood G (*G*-statistic = 79.298, p = 0.18) but remained significant based on *F*_ST_ (*F*_ST_ = 0.012; p = 0.009). Overall this suggests very low population differentiation between colonies.

**Table 2:** Population differentiation from *F*_ST_ values (above diagonal) and log likelihood G-statistic values (below diagonal) for all pairwise comparisons.

	Pop1	Pop2	Pop3
Pop1	—	0.003	0.013*
Pop2	100.007	—	−0.005
Pop3	136.214*	83.901	—

Significant differences after Bonferroni correction (0.05/3) are noted with *. However, excluding the three loci with deviations from HWE results in no pairwise population comparison being significant.

### Spatial genetic structure

**Figure 3:**
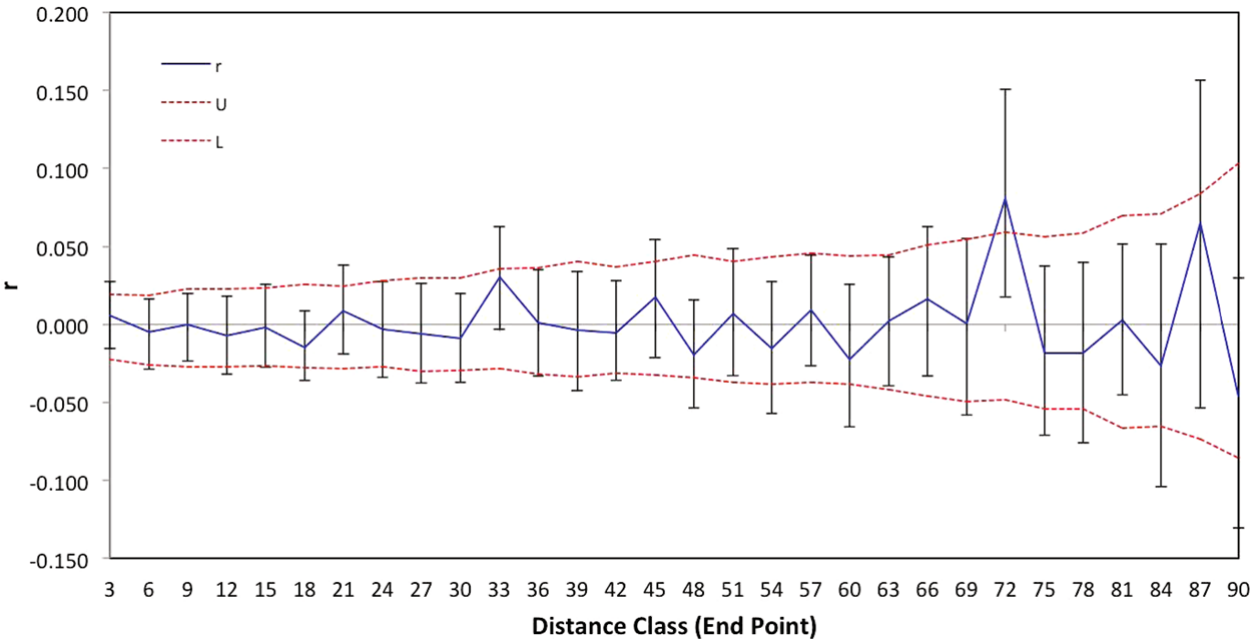
Correlogram of the linear spatial genetic relationship (*r*) among individuals within colonies, assessed at 3 m distance classes. Upper and lower error bars bound the 95% confidence interval for *r* as determined by bootstrap resampling. Upper and lower confidence limits (dashed lines) bound the 95% confidence on the null hypothesis of no spatial genetic structure.

No spatial genetic structure was detected within colonies. Inter-individual differentiation at all distance classes was non-significant except at 72 m intervals (bias corrected *r* = 0.081, p < 0.003) but as this is only one distance class at high linear distance we do not consider it biologically significant. Importantly, in all other distance classes, *r* falls within the upper and lower bounds and the error bars on *r* always include 0 (Fig. 3).

The test for heterogeneity, which would indicate a deviation of spatial genetic correlation relationships across all colonies, is non-significant (combined correlogram omega = 88.58, p = 0.015 when p < 0.01 is significant). When distance classes are reduced to 15, to only include the maximum class at which there are data from all 3 colonies (as the linear length of colonies differs), there is still no evidence for spatial genetic autocorrelation (omega = 37.89, p = 0.16).

### Sex-biased dispersal

We found no evidence for sex biased dispersal in *A. astorquii*, reflected as no significant difference between the mean AIc (Assignment Index correction) values for males vs. females in assignment tests. Both male AIc = 0.190 ± 0.275 and female AIc = −0.172 ± 0.234, (mean ± SE) bounded zero, and no difference between the two sexes was found (U-test of difference between males and females: two tailed probably for |Z| = 0.211, non-significant) (Fig. S2).

## Discussion

The scope of this study was to assess within and between colony population genetic structure in *A*. *astorquii*, a colony-breeding cichlid fish living in Nicaragua’s largest crater lake, Lake Apoyo. The results show no spatial genetic structure within the three breeding *A*. *astorquii* colonies, contrary to what might have been predicted under site fidelity or kin-clustering. Similarly, there was little or no population differentiation among the colonies; the lake is effectively a panmictic population. Furthermore, there was no sex-bias to this lack of genetic structure. The lack of genetic differences among colonies suggests that there is no colony fidelity in *A*. *astorquii*. Either adult individuals commonly settle into different breeding colonies for different breeding seasons or, after becoming independent, juveniles disperse away from their natal colony. In other words, the seasonal aggregations of breeders may temporally alternate with more widely ranging life-history phases. In this respect, the results are similar to many generalist and pelagic cichlid species^4,12,13^, including *A*. *zaliosus* from Lake Apoyo^38^ and *Amphilophus xiloaensis* from nearby crater lake Xiloá^59^ that were not found to show any significant population structure across different sites within the lakes. However, our results are in contrast with previous studies on African cichlids that have shown that benthic (and other specialised) species tend to have moderate to high levels of spatial genetic structuring, including high fidelity to breeding grounds and an inhibition to crossing habitats^3,4,12,60–62^.

Because more pronounced geographic structure has been found in fish at scales much smaller than the size of Lake Apoyo^3,4,61^, the size of the lake alone cannot explain the observed lack of population structure in these cichlids. One potential explanation for this difference could be fluctuations in the existence and distribution of *Chara* algae beds where all *A*. *astorquii* breeding grounds were located at the time of this study (Fig. 1). In particular, the occurrence of *Chara* in this lake varies over time^63^ (personal observations 2007–2016), and hence the location of breeding aggregations associated with *Chara* may not remain stable enough for philopatry to evolve or for any population structuring to build up detectable genetic signal. This hypothesis is also in line with our finding that there was no sex-bias to the lack of structure between or within the colonies, while earlier studies on African cichlids have suggested that dispersal in cichlids can be either male^15,17,64^ or female^65^ biased.

*Amphilophus astorquii* belongs to the extremely rapidly speciating lineage of cichlid fish, and it has speciated sypatrically within a few hundred generations after the formation of Lake Apoyo (approximately 23000 years ago^44^) within the lake^39,46^. Our results are therefore relevant also in the context of inferring the most likely modes of rapid speciation taking place within the lake. The comparison between colonies from different parts of the lake suggests that there is no biologically significant level of population structure within the species. This makes the occurrence of parapatric speciation via strong ‘homing’ unlikely^30^. Indeed, together with the previous studies that found no significant population structure within other Nicaraguan crater lake cichlids^38,56^, the current study provides evidence against the existence of barriers to gene flow within the lake. The lack of such barriers is in accordance with the proposition of sympatric speciation being important in the origin of these species^36,38,39^. Other modes of speciation, e.g. through disruptive natural selection, have been proposed to explain elevated speciation rates in taxa showing less genetic population-structure^66–68^ and are also likely to play an important role in the (sympatric) origin of cichlid species in Nicaraguan crater lakes, such as Lake Apoyo^38,39,69^.

To conclude, we found no population structure in *A*. *astorquii* either at the colony or lake-wide level. The results are important in suggesting that strong philopatry or sex biased dispersal are unlikely to contribute markedly to the rapid speciation process the species has undergone within Lake Apoyo, providing further support for sympatric ecological speciation as the driver of the extremely rapid diversification of these cichlids in Nicaraguan crater lakes in Nicaragua.

## Electronic supplementary material

Supplementary materials
