# Supplementary material for: Genetic evidence for panmixia in a colony-breeding crater lake cichlid fish
Source: Sci Rep. 2018 Jan 18;8:1166. doi: 10.1038/s41598-018-19266-5 (PMC5773479; doi:10.1038/s41598-018-19266-5)
Supplement: Supplementary file 1 — Supplementary materials [file 41598_2018_19266_MOESM1_ESM.doc]

**Genetic evidence for panmixia in a colony-breeding crater lake cichlid fish**

T. K. Lehtonen^1,2,*^, K. R. Elmer^1,3,*^, M. Lappalainen^3^ & A. Meyer^1^

^1^ Zoology and Evolutionary Biology, Department of Biology, University of Konstanz, 78457 Konstanz, Germany

^2^ School of Biological Sciences, Monash University, Victoria 3800, Australia

^3^ Institute of Biodiversity, Animal Health & Comparative Medicine, College of Medical, Veterinary & Life Sciences, University of Glasgow, Glasgow, G12 8QQ, UK

^*^ Equal contributors

Correspondence:

axel.meyer@uni-konstanz.de

Tel. +49 7531 884163

Fax +49 7531 883018

Table S1. Summary diversity statistics of all populations combined. Standard deviations were obtained through jackknifing over loci. 95% confidence intervals were obtained through bootstrapping over loci.

|  | **Value** | **± S.D.** | **c.i. 2.5%** | **c.i. 97.5%** |
| --- | --- | --- | --- | --- |
| Number of alleles (Num) | 8.818 | 1.32 | 6.455 | 11.273 |
| Effective number of alleles (Nae) | 2.982 | 0.473 | 2.127 | 3.899 |
| Observed Heterozygosity (Ho) | 0.510 | 0.073 | 0.37 | 0.643 |
| Heterozygosity Within Populations (Hs) | 0.576 | 0.073 | 0.426 | 0.702 |
| Total Heterozygosity (Ht) | 0.577 | 0.073 | 0.428 | 0.703 |
| Corrected total Heterozygosity (H't) | 0.578 | 0.073 | 0.429 | 0.703 |

Table S2. For each colony as a population, per locus Weir & Cockerham *F*_IS_ estimate and two-sided p-value significance after Bonferroni correction (*=p < 0.017) is listed. Multilocus significance based on Fisher’s Chi2 method (* = highly significant).

|  |  |  |  |  |  |  |  |  |  |  |  |  |
| --- | --- | --- | --- | --- | --- | --- | --- | --- | --- | --- | --- | --- |
|  | **Abur28** | **Abur45** | **Abur82** | **Abur151** | **M1M** | **M2** | **M7** | **M12** | **TmoM7** | **Unh012** | **Unh013** | **Multi-locus** |
| Colony 1 | 0.20 | 0.12* | -0.04 | 0.09 | -0.02 | 0.05 | 0.05 | 0.08 | 0.03 | 0.40* | 0.00 | * |
| Colony 2 | 0.21* | 0.31 | -0.07 | -0.25 | -0.03 | -0.01 | 0.06 | 0.29* | 0.30 | 0.25* | -0.06 | * |
| Colony 3 | 0.06 | 0.70* | -0.03 | -0.10 | -0.01 | 0.16 | -0.11 | -0.31 | 0.49 | 0.49* | 0.04 | * |

Table S3. Genotypes, sample location, and sex for n = 123 adult *Amphilophus astorquii* at 11 loci. Missing data are coded with 0. Column 'Pop' refers to the 3 colonies and column 'x' shows the placement within the colonies (as linear distance in metres).

Figure S1. Log likelihood (blue circle) ± standard deviation (grey line) from Structure analysis of genetic clustering assessing one to four groups.


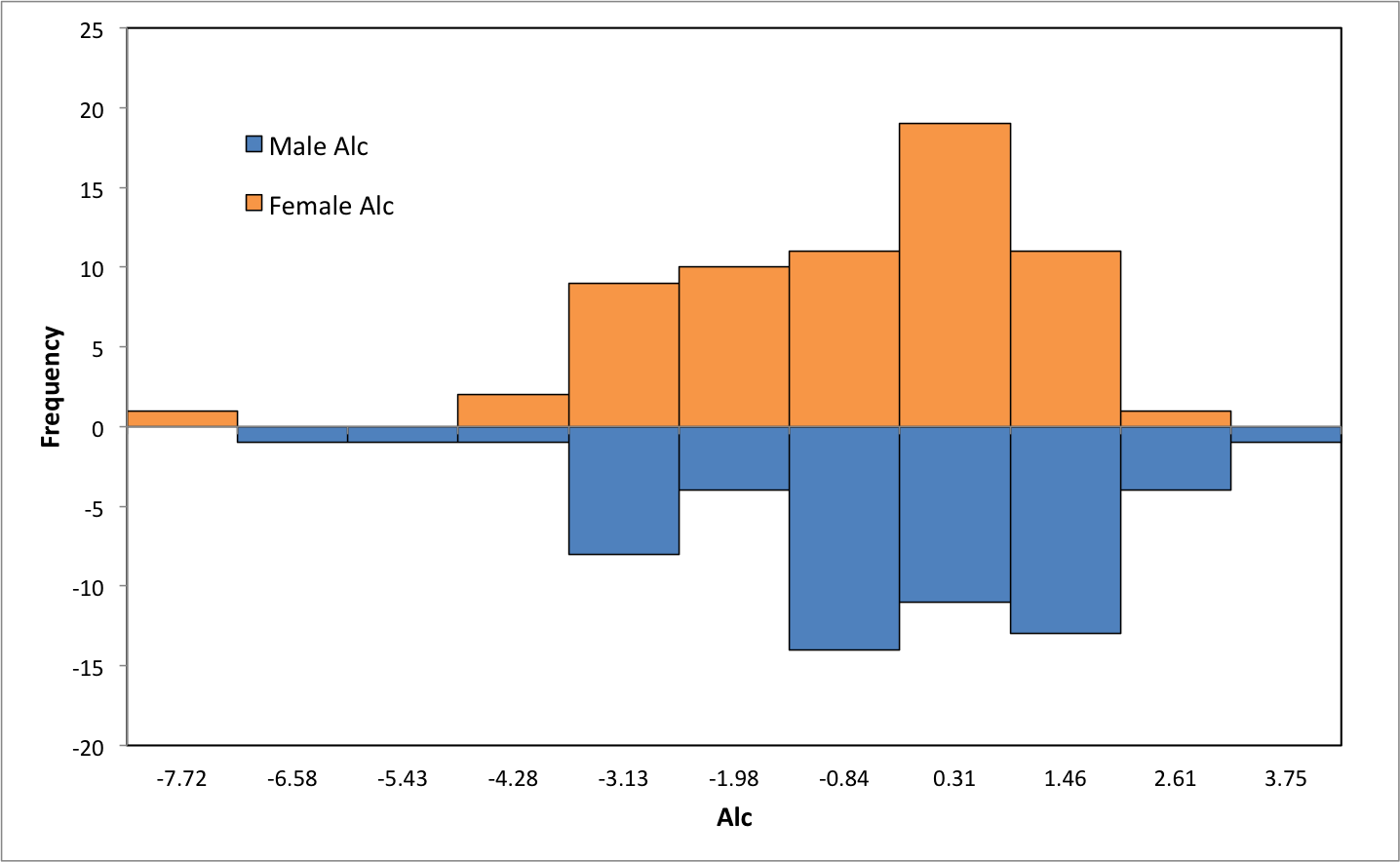


Figure S2. The frequency distribution of assignment indices (AIc) for males (below axis) and females (above axis) of the *A*. *astorquii* population in Lake Apoyo.
